# Tuberculosis in Cold-Blooded Animals

**Published:** 1898-04-23

**Authors:** 


### TUBERCULOSIS IN COLD-BLOODED ANIMALS.

The fact has been so often driven in upon us that tuberculosis is especially fostered by confinement and by an unnatural mode of life that, perhaps, any additional illustration of such a platitude is hardly worth the seeking. The announcement, however, that tuberculosis has been communicated to certain carp confined in a fish-pond is of some interest outside and beyond the general confirmation it affords to widely-accepted views; for some change seems to have come over the tubercle bacilli engaged in giving these fishes this disease. Their virulence has in some way become so attenuated by cultivation in these colder-blooded creatures that, while they have retained their power of transmitting the disease to other fishes, they have so lost their virulence towards warm-blooded animals that the disease cannot be transmitted back again to them. The analogy with certain other facts in experimental and comparative pathology is full of interest.

